# Potatoes as Pen Wipers

**Published:** 1883-07

**Authors:** 


### POTATOES AS PEN-WIPERS.

A writer in a German paper says that it is the custom in offices in that country to have a sliced potato on the desk for use as a pen-wiper, and to clean steel pens. It removes all ink crusts, and gives a peculiar smooth flow to ink. New pens should be passed two or three times through the gas flame to remove the grease with which they are coated. The ink will then flow freely.

